# PanStop: a decade of rapid containment exercises for pandemic preparedness in the WHO Western Pacific Region

**DOI:** 10.5365/wpsar.2018.9.5.012

**Published:** 2018-12-18

**Authors:** Edna Moturi, Katherine Horton, Leila Bell, Lucy Breakwell, Erica Dueger

**Affiliations:** a WHO Regional Office for the Western Pacific, Manila, Philippines.; b Influenza Division, Centers for Disease Control and Prevention, Atlanta, United States of America.

Rapid containment (RC) is one of the five priority interventions of the World Health Organization (WHO) Strategic Action Plan for Pandemic Influenza; (*1*) it relies on the concept that mass prophylactic administration of antiviral drugs, combined with quarantine and social distancing measures, could contain or delay the international spread of an emerging influenza virus. (*2*, *3*) During a RC operation, mass antiviral prophylaxis treatment and non-pharmaceutical interventions are rapidly implemented within a containment zone surrounding the initial cases; active surveillance and additional activities are extended to a broader buffer zone where cases are most likely to appear based on the movements of cases and contacts. (*2*, *4*) The strategy is dependent on the rapid (within three to five days) detection, investigation and reporting of initial cases; the efficacy and availability of antivirals and vaccines; and timely risk assessment and decision-making. In the Western Pacific Region, a stockpile of antiviral medication and personal protective equipment acquired through donations from the Government of Japan is warehoused in Singapore under the auspices of the Association of South-eastern Asian Nations (ASEAN), (*5*) and is managed under contract by the Japan International Cooperation System (JICS). (*5*) These supplies are reserved for early intervention when initial signs of increased human-to-human transmission of a highly contagious influenza virus occur.

Advanced planning is required for RC to ensure that all relevant partners and sectors work together in a coordinated manner within a short time frame. Simulation exercises are recognized as a crucial component of pandemic preparedness, and many different types of exercises have been conducted in the Asia–Pacific region. (*6*) In this paper, we describe the PanStop exercises conducted by the WHO Regional Office for the Western Pacific to show how they have contributed to pandemic preparedness in the Region.

In 2007, the WHO Regional Office for the Western Pacific conducted the first simulated exercise known as PanStop that aimed to determine the validity of procedures developed in the Asia–Pacific Region for RC of a new, highly contagious influenza virus. (*5*) The exercise took 11 hours over one and a half days, and was conducted in six sites, at the WHO Regional Office for the Western Pacific, the WHO Country Office in Cambodia, JICS and the Japanese Ministry of Foreign Affairs in Tokyo, the ASEAN Secretariat in Jakarta, and the offices of Singapore Technologies Logistics (STL) in Singapore. (*5*) Since then, the WHO Regional Office for the Western Pacific has conducted nine PanStop exercises on RC to identify strengths and opportunities to improve planning activities for containing pandemic influenza. PanStop is designed to test pandemic influenza response plans through a simulated real-world event and is not designed to evaluate individual participant performance.

PanStop exercises typically involve artificial but realistic scenarios where human infections of a novel influenza A virus are reported from a Member State. Participants, who may include WHO staff, ministry of health officials and people from other government agencies potentially involved in pandemic response, work through and discuss the decision-making process and actions necessary to implement RC based on their current pandemic preparedness plans. Each year, Member States or WHO country offices may request that a PanStop exercise be conducted in their country to test their levels of preparedness. The Regional Office has also been the main player in two exercises to test and evaluate the roles and responsibilities of Regional Office staff for regional RC, particularity in logistics and communication.

Both modified functional and table-top exercises have been employed for PanStop exercises (**Table 1**). A modified functional exercise is an interactive process where multisectoral participants receive simulated outbreak information through e-mail, telephone or actions and then respond as they would within actual designated roles. Participants may carry out tasks in response to outbreak information (e.g. prepare a line list of cases, develop talking points for a press conference, calculate required doses of prophylaxis) or, when time is constrained, they may be asked to describe the actions they would take. These functional exercises have been conducted in Cambodia (2007), (*5*) the Philippines (2008), (*7*) Malaysia (2009), (*8*) Mongolia (2010), (*9*) Viet Nam (2013), (*11*) and at the WHO Regional Office of the Western Pacific (2011), (*10*) 2014 (*12*) and 2015 (*13*) (**Table 1**). All the exercises involved fictional scenarios of diseases of unknown etiology or occurrence of novel avian influenza with evidence of human-to-human transmission which necessitated the launch of a RC exercise. With the exception of PanStop 2007 and 2010, all the exercises were conducted within two days. PanStop exercises conducted at the WHO Regional Office of the Western Pacific have typically included JICS and ASEAN to test their transportation protocols when they ship items in the regional stockpile from Singapore to the requested country.

**Table 1:** PanStop exercises on influenza pandemic responses in the Western Pacific Region, 2007–2018

Year	Country	Objectives	Scenario summary	Type of exercise	Duration	Participating agencies	Lessons learnt
**2007**	WHO Regional Office and Cambodia (*5*)	Practise decision-making and communication with partner agencies to launch and manage a RC operation; train staff in RC; develop a replicable model exercise for training other jurisdictions.	Fictional discovery of cases of novel strain of avian influenza in a village with evidence of human-to-human transmission.	Modified functional	1½ days	WHO Regional Office, WHO Country Office in Cambodia, ASEAN, JICS, STL, Cambodia Ministry of Health, and the Cambodian National Centre for Disaster Management	It is safer to be proactive and deploy resources in waves, despite the consequences of lacking data, than to respond too late.
**2008**	Philippines (*7*)	Assess the preparedness of the Philippines to implement a RC operation; gain a better understanding of operational capacity for RC in the country.	Fictional outbreak of a potential pandemic strain of the avian influenza virus in the Philippines.	Modified functional and table-top	2 days	WHO Regional Office, JICS, ASEAN, Philippine Department of Health, and various agencies and authorities, including the Armed Forces and Police	Philippine Government and nongovernmental agencies now understand a RC operation from a national perspective.
**2009**	Malaysia (*8*)	Identify strengths and opportunities for improvement in planning activities for pandemic influenza; gain better understanding on the operational capacity for RC activities in Malaysia.	Fictional outbreak of a potential pandemic strain of avian influenza in a village in Johor State.	Modified functional	2 days	WHO Country Office in Malaysia, WHO Regional Office and various health and disaster management ministries and authorities	Ministry of Health to take lead in strengthening the management processes or emergency operations at state, district and field levels.
**2010**	Mongolia (*9*)	Test WHO decision-making processes during a routine rapid response and before launching a RC operation.	Fictional outbreak of a potentially pandemic strain of influenza virus in a district in Mongolia.	Modified functional	Six hours	WHO Country Office in Mongolia, WHO Regional Office, Ministry of Foreign Affairs, Japan, JICA	Exercise led to a deepened understanding of RC protocol and identified need to clarify stakeholders’ roles and responsibilities in the RC protocol.
**2011**	WHO Regional Office and, Philippines (*10*)	Test the responses of the WHO Regional Office during RC in a Member State to evaluate roles and responsibilities of response logistics, validate risk communication; assess operational issues and processes to establish, maintain and close the containment zone.	Fictional outbreak of a disease occurring in a hypothetical country in WPR.	Functional table-top	2 days	WHO Country Office in the Philippines, WHO Regional Office, JICS	There was a need for a RC plan to serve as an outline for planning future exercises. High staff turnover requires frequent training exercises.
**2013**	Viet Nam (*11*)	To practise and strengthen processes within the health ministry in Viet Nam before a decision to initiate a RC for an outbreak of influenza with pandemic potential.	Fictional outbreak of a disease of unknown etiology in a northern province in Viet Nam.	Modified functional	2 days	Viet Nam Health Ministry, WHO Country Office for Viet Nam, JICS, ASEAN, Asia-Europe Foundation, CDC	Health ministry should take the lead in developing guidelines, decision-making tools and RC logistics plans, including those to be involved in the process.
**2014**	WHO Regional Office (*12*)	For WHO staff to become familiar with RC decision-making process and to understand their RC roles.	Fictional outbreak of a novel avian influenza virus in a hypothetical country.	Modified functional	2 days	WHO Regional Office, JICS	There is a need for expanded guidance for RC, training on the role of JICS in RC, and improvement to EOC activation plans.
**2015**	WHO Regional Office (*13*)	For WHO staff to evaluate the need for RC and initiate an operation; provide training on roles and responsibilities within an active EOC as developed in previous PanStop exercise.	Fictional outbreak of a novel avian influenza virus in a hypothetical country.	Modified functional	2 days	WHO Regional Office, JICS	RC briefing and training documents should be updated and expanded, EOC procedures maintained and validated, and status board templates developed.
**2017**	Fiji (*14*)	For health ministry and partner staff to evaluate roles, responsibilities and decision-making for a RC operation, including response logistics and risk communication.	Fictional outbreak of a novel avian influenza virus in a district in Fiji that escalates into a national emergency.	Table-top	2 days	WHO Country Office in Fiji, WHO Regional Office, various ministries and authorities, including health, police and military	Strengthened multisectoral collaboration is key to the success of a RC operation.
**2018**	Mongolia	For health ministry and partner staff to evaluate roles, responsibilities and decision-making for a RC operation, including response logistics and risk communication.	Fictional outbreak of a novel avian influenza virus in Choibalsan city.	Table-top	1½ days	WHO Country Office in Mongolia, WHO Regional Office, and various health and disaster management ministries and authorities	Exercise identified a need for improved coordination within the health sector and promotion of intersectoral preparedness and response planning.

ASEAN: Association of South-eastern Asian Nations; CDC: Centers for Disease Control and Prevention; EOC: Emergency Operations Centre; JICA: Japan International Cooperation Agency; JICS: Japan International Cooperation System; RC: rapid containment; STL: Singapore Technologies Logistics; WHO: World Health Organization.

A table-top exercise comprises the same stakeholders, but a facilitator guides a discussion about a simulated series of events that prompts discussion of response actions from participants. Table-top exercises provide an opportunity for moderated interactions of multiple sectors in addition to the ministry of health. In 2017, a table-top PanStop exercise was held in Fiji at the request of the Fiji Ministry of Health to test the readiness of organizations involved in the national pandemic preparedness plan, including ministries of agriculture and transportation. (*14*) The exercise, which lasted one and a half days, highlighted the importance of good multisectoral collaboration in ensuring a successful response. A similar table-top exercise was conducted this year in Mongolia involving a fictional outbreak of novel avian influenza A(H10N8) in Choibalsan province with multisectoral participation from the WHO Regional Office, WHO Country Office in Mongolia, JICS and various ministries and authorities.

As for all simulation exercises, PanStop is a relatively inexpensive way of assessing operational readiness and is more feasible than full-scale exercises that require extensive financial and human resource investment. PanStop exercises typically last one or two days with simulated deployment of human and physical resources rather than actual movement of these resources. The exercises provide an opportunity for multisectoral engagement as RC requires involvement from both animal and human health sectors as well many other stakeholders, including administration, communication and logistics specialists (**Table 1**). PanStop exercises are overseen by evaluators who are pandemic preparedness experts. They assess the participants’ actions in terms of their appropriateness and alignment with the exercise’s goals and objectives. Through consultation with participants, they also recommend improvements for operational readiness for RC. A final report is published for each conducted PanStop exercise that includes the evaluation results, lessons learnt and recommendations (**Table 1**).

Lessons learnt from PanStop exercises include the need to (1) update national pandemic preparedness plans; (2) clarify specific sector roles during both RC and pandemic response efforts; (3) emphasize concepts to senior officials from different government agencies that may be involved in pandemic response; and (4) allow stakeholders to identify knowledge and planning gaps, such as lack of standardized operating procedures for RC initiation and availability of trained staff to execute the plans. A lesson learnt from the exercise at the regional level in 2014 was the need to improve Emergency Operations Centre activation plans. As a result, the improved plans were developed, implemented and successfully tested in the 2015 PanStop exercise (**Table 1**). Recommendations for improvements to the PanStop exercise have been made so that future exercises are more effective and can potentially evolve beyond RC to test broader national systems.

Many national governments within the Western Pacific Region have developed national pandemic response plans for RC to prepare for the next influenza pandemic. (*15*) It is critical that these plans have the ability to be operationalized efficiently to mitigate the consequences of the next pandemic. PanStop exercises provide an opportunity to test the RC mechanisms of these pandemic plans in the participating countries and at the regional level in a simulated environment that imitates pandemic events as they unfold. By participating in these exercises and subsequently adapting national preparedness plans based on exercise outcomes, the readiness capacity of participating governments, WHO and other partners in the Region improves for the next influenza pandemic.
